# Effects of elevated carbon dioxide on growth, feed efficiency and physiological responses in RAS-based juvenile pikeperch (*Sander lucioperca*)

**DOI:** 10.3389/fvets.2026.1812125

**Published:** 2026-04-13

**Authors:** Tomáš Pěnka, Václav Kučera, Laura Ostolaza Perez-Cruz, Oleksandr Malinovskyi, Jitka Kolárová, Uroš Ljubobratović, Tomáš Policar

**Affiliations:** 1 Faculty of Fisheries and Protection of Waters, South Bohemian Research Center of Aquaculture and Biodiversity of Hydrocenoses, University of South Bohemia in České Budějovice, Vodnany, Czechia; 2 Research Center for Aquaculture and Fisheries, Institute of Aquaculture and Environmental Safety, Hungarian University of Agriculture and Life Sciences, Szarvas, Hungary

**Keywords:** carbon dioxide, fin condition, growth performance, hypercapnia, intensive aquaculture, pikeperch

## Abstract

Carbon dioxide (CO_2_) accumulation is a major concern in recirculating aquaculture systems (RAS), where elevated levels can negatively affect fish growth and welfare. Although the effects of CO_2_ on pikeperch (*Sander lucioperca*) have been studied, long-term tolerance under production conditions is not well documented. This study investigated the effects of three concentrations of dissolved CO_2_ (5 mg L^−1^ = Low; 15 mg L^−1^ = Medium; 30 mg L^−1^ = High). On the growth performance, conditional parameters, and physiological status of juvenile pikeperch reared for 105 days in RAS. Fish (initial total length 250.9 ± 20.7 mm; initial weight 117 ± 28.2 g) were stocked in three tanks for treatment and fed at 1% biomass. Fish in the High group showed significantly lower final weight (233 g) than fish in the Low (297 g) and Medium (250 g) groups, correlated by a reduced Fulton's condition factor and a significant increase in feed conversion ratio (1.09–1.82). Other production parameters, survival rate, organosomatic indices, fin erosion did not show significant differences between groups. Biochemical parameters highlighted stress and reduced protein metabolism under high CO_2_. Total plasma protein, albumin and globulin were significantly lower in the High group, while plasma ammonia and cortisol increased significantly in group High. These results indicate that CO_2_ concentrations above 15 mg L^−^1 impair growth, feeding efficiency and physiological condition of juvenile pikeperch, highlighting the need for strict CO_2_ control in RAS.

## 1 Introduction

Recirculating aquaculture systems (RAS) enable high production with constrained water use, but dissolved carbon dioxide (CO_2_) can accumulate and chronically challenge fish physiology and performance. Building on prior work across percids and salmonids, chronic hypercapnia is linked to reduced feed intake and integumentary changes under production-relevant conditions (1, 2). Elevated CO_2_ interacts with pH and alkalinity, disrupting acid–base balance and appetite, and increasing risk of nephrocalcinosis in RAS-reared fish (3–6). Current guidelines generally recommend maintaining CO_2_ below 15–20 mg L^−1^ for most teleosts, but species-specific and life-stage tolerances vary considerably, and thresholds derived from laboratory trials can underestimate risks under production-scale conditions (7, 8).

Pikeperch (*Sander lucioperca*) is increasingly recognized as a high-value species for European inland aquaculture due to its market demand and compatibility with land-based systems (9–12). In previous study (8), the adult pikeperch have demonstrated linear declines in growth and physiological adjustments (including reduced hematocrit and altered oxygen consumption), when exposed to CO_2_ concentrations ranging from 4 to 30 mg L^−1^. These findings underscore the sensitivity of pikeperch to hypercapnia. However, this conclusion is limited by short experimental durations, laboratory-scale conditions, and focus on older life stages (13). This creates a critical knowledge gap regarding long-term CO_2_ tolerance in juvenile pikeperch under realistic production settings, where cumulative stressors and system chemistry may exacerbate physiological challenges (6, 14, 15).

From a mechanistic perspective, elevated environmental CO_2_ triggers respiratory acidosis; teleosts partly compensate via branchial ion exchange (Na^+^/H^+^ and Cl^−^/${\text{HCO}}_{3}^{-}$) supported by systemic bicarbonate buffering (16, 17). Compensation is often incomplete at higher carbon dioxide (pCO_2_), so extracellular pH and aerobic performance may remain depressed, ultimately constraining growth and condition (16, 18, 19). Recent reviews highlight that chronic CO_2_ exposure triggers transcriptional changes and interact with osmoregulatory processes, reinforcing the importance of robust degassing and continuous CO_2_ monitoring in RAS (6, 20).

Beyond percids, research on Atlantic salmon post-smolts in brackish RAS has shown significantly reduced growth and integumentary changes at CO_2_ levels ≥12 mg L^−1^ compared to 5 mg L^−1^, without overt nephrocalcinosis (7, 21, 22). Analyses confirm that elevated CO_2_ consistently depresses feed intake and FCR efficiency across taxa, emphasizing the economic and welfare implications of inadequate CO_2_ control (21, 22). These findings collectively highlight the need for species and life-stage specific thresholds under commercial conditions.

Unlike previous study (8), this experiment evaluates juvenile pikeperch over a prolonged 105-day period in production-scale RAS, integrating growth performance, feed efficiency, somatic indices, and plasma biochemistry across three CO_2_ regimes (5 mg L^−1^ = Low; 15 mg L^−1^ = Medium; 30 mg L^−1^= High). We tested at 5, 15, and 30 mg L^−1^, which correspond to: (i) a low, near-ambient level for well-degassed RAS (5 mg L^−1^); (ii) a level near the upper bound of a range of concentrations that are within the generally recommended operating range for many teleosts (15–20 mg L^−1^); (iii) a level that is near a maximum, yet operational, concentration (30 mg L^−1^), which may be found at high biomass densities and suboptimal degassing. These concentrations span a relevant ecological range for production-scale RAS, are consistent with published recommendations and empirical data for a range of species and directly investigate performance near and above the practical threshold for juvenile pikeperch (2, 6–8, 21–23). By simulating realistic operational conditions, this study provides critical insights into CO_2_ tolerance during early ongrowing (a phase pivotal for economic viability) and informs best practices for welfare-optimized culture.

## 2 Materials and methods

### 2.1 Ethical approval

All experimental procedures in this study complied with the applicable animal welfare legislation of the Czech Republic. Ethical approval was obtained from the Animal Welfare Committee of the Laboratory of Intensive Aquaculture (LIA), Faculty of Fisheries and Protection of Waters (FFPW), University of South Bohemia in České Budějovice (USB), Czech Republic. All procedures were conducted in accordance with national legislation, specifically Act No. 166/1996 Coll. on Veterinary Care and Act No. 246/1992 Coll. on the Protection of Animals Against Cruelty. Additionally, the study was authorized by the Departmental Expert Committee for the Authorization of Experimental Projects under the Ministry of Agriculture of the Czech Republic (protocol number: MZE-39723/2025-13143; MZE-61035/2024).

### 2.2 Experimental setup of RAS

The 105-day trial was conducted in the three experimental RAS at the Laboratory of Intensive Aquaculture (LIA), Faculty of Fisheries and Protection of Waters, University of South Bohemia (Vodnany, Czech Republic). Each system was identical and previously described in several published studies (24–27), comprises multiple integrated components, including 15 light-gray cylindrical plastic tanks (diameter: 885 mm; height: 620 mm; water volume: 380 L), a mechanical filter (Ratz Aqua & Polymer Technik GmbH, Remscheid, Germany), and a two-stage biological filtration system. The first stage consists of a custom-designed moving bed filter (volume: 890 L), while the second stage employs a commercial Nexus 310 filter (Evolution Aqua Ltd., Wigan, UK; volume: 100 L).

Aeration for both filters was provided by a Secoh EL-S 250 W air blower (Secoh Shanghai Mec. Ltd., Shanghai, China), with air distributed via air stones and plastic grates positioned at the bottom of the filters. These components ensured efficient mechanical and biological filtration. Oxygen consumption within the system ranged from 1.50 to 4.00 L min^−1^, increasing exponentially with fish growth and biomass.

A controlled photoperiod of 12 h light (06:00–18:00) and 12 h dark was maintained, with low light intensity (seven lux on the water surface), measured using a UNITEST 93514 luxmeter (Beha-Amprobe GmbH, Glottertal, Germany). Water temperature and dissolved oxygen were monitored three times daily (07:00, 14:30, and 18:00) using a YSI ProODO oximeter (YSI Inc., Yellow Springs, OH, USA). Daily monitoring also included pH measurements (WTW 3310 pH meter; Xylem Ceská republika, spol. s r.o., Prague, Czech Republic) (28). The concentrations of ammonium nitrogen (${\text{NH}}_{4}^{+}$–N) and nitrite nitrogen (${\text{NO}}_{2}^{-}$–N) were quantified using titration-based colorimetric test kits in accordance with standard analytical procedures. Ammonium levels were assessed with a Nessler method, employing Nessler's reagent (K_2_[HgI_4_]; Merck Life Science spol. s r. o., Prague, Czech Republic) following prior complexation with Seignette salt (KNaC_4_H_4_O_6_·4H_2_O; PENTA s.r.o., Prague, Czech Republic). Nitrite determination was performed via the Griess reaction, which utilizes sulfanilic acid (C_6_H_7_NO_3_S) in combination with N-(1-naphthyl)ethylenediamine dihydrochloride (NED; PENTA s.r.o., Prague, Czech Republic). The applied kits provided detection ranges of 0.02–2.0 mg L^−1^ for ${\text{NH}}_{4}^{+}$-N and 0.01–1.0 mg L^−1^ for ${\text{NO}}_{2}^{-}$-N. The obtained values were subsequently recalculated to total ammonia and total nitrite using conversion coefficients derived from calibration datasets maintained by the Laboratory of Aquatic Toxicology and Ichthyopathology at the FFPW (Vodnany, Czech Republic). To maintain stable and high-water quality, 5% of the system volume was replaced daily with tap water, and a flow rate of 3 m^3^ h^−1^ was applied, ensuring complete water turnover twice per hour in each experimental tank (25).

Dissolved CO_2_ was measured three times daily (07:00, 14:30, and 18:00) using the OxyGuard CO_2_ portable analyzer G03C2 (OxyGuard^®^ International A/S, Farum, Denmark). For clarity of environmental context and consistency of exposure conditions, the group-wise mean ± standard deviation (SD) values for key water-quality variables (dissolved CO_2_, pH, O_2_ saturation, temperature, ${\text{NH}}_{4}^{+}$, ${\text{NO}}_{2}^{-}$) are presented in Table 1.

**Table 1:** Average values selected water chemistry parameters for each RAS unit after 105 days (15 weeks) for CO_2_ groups: 5 mg L^−1^ = low; 15 mg L^−1^ = medium; 30 mg L^−1^= high.

Parameter	Low	Medium	High
Dissolved CO_2_ (mg L^−1^)	4.95 ± 2.17	14.67 ± 4.70	29.50 ± 5.08
pH	7.04 ± 0.15	6.94 ± 0.53	6.88 ± 0.20
Oxygen saturation (%)	107.16 ± 7.15	104.56 ± 6.28	108.50 ± 9.14
Temperature (°C)	22.06 ± 0.31	22.19 ± 0.68	21.88 ± 0.53
${\text{NH}}_{4}^{+}$ (mg L^−1^)	0.56 ± 0.29	0.61 ± 0.38	0.66 ± 0.40
${\text{NO}}_{2}^{-}$ (mg L^−1^)	0.59 ± 0.18	0.76 ± 0.23	0.89 ± 0.36

Values expressed as mean ± SD.

${\text{NH}}_{4}^{+}$, ammonium cation; ${\text{NO}}_{2}^{-}$, nitrite anion; pH, logarithmic and inversely indicates the activity of hydrogen cations in the solution: pH = –log_10_ ([H^+^]/M).

### 2.3 Dissolved CO_2_ enrichment

The experiment was conducted using three independent RAS units, each containing three replicate tanks (a total of nine tanks). These units were assigned to three dissolved CO_2_ regimes for 105 experimental days (15 weeks): 5 mg L^−1^ (Low), 15 mg L^−1^ (Medium), and 30 mg L^−1^ (High). To maintain target concentrations, food-grade CO_2_ (Linde Gas a.s., Prague, Czech Republic) was pumped into the high and medium regime RAS units using modular Wedge Lock diffusers (Pentair Aquatic Eco-Systems, Apopka, FL, USA) installed in the pump sumps (so that the water was evenly distributed in the tanks). CO_2_ pumping and flow to the RAS units (Medium and High) were controlled independently using separate CO_2_ pressure cylinders (Linde Gas a.s., Prague, Czech Republic), each equipped with a cylinder pressure regulator RV CO2-10 bar-G3/4”-G $\frac{1}{4}$” (Linde Gas a.s., Prague, Czech Republic). Before the experiment began, 3 weeks of pre-testing were conducted to ensure stable dissolved CO_2_ concentration in the RAS in tested groups (Medium and High) with using the OxyGuard CO_2_ portable analyzer G03C2 (OxyGuard^®^ International A/S, Farum, Denmark). The third RAS unit (Low) received no supplemental CO_2_ and served as low regime control.

### 2.4 Fish experimental groups

The juvenile pikeperch used in this experiment were reared using a combination of intensive and pond-based aquaculture techniques in the LIA (Vodnany, Czech Republic) (29, 30). All experimental fish were 4 months old with the comparable initial body weight at the start of the experiment.

At the beginning of experiment, a total of 450 juveniles pikeperch with initial total length (iTL) = 250.9 ± 20.7 mm (mean ± SD), initial standard length (iSL) = 218.9 ± 16.9 mm and initial body weight (iBW) = 117.7 ± 28.2 g were distributed among three experimental RAS.

Fifty experimental fish were stocked (into each tank resulting) in an initial stocking density of 15.5 kg m^−3^. Finally nine tanks in three RAS were used, when each RAS contains three tanks presenting each tested CO_2_ level. Fish were fed during the day from 8:00 a.m. to 4:00 p.m., i.e., a total of 8 h a day using belt feeders with a commercial floating diet [Biomar EFICO 7170F, with diameter 3 mm (BioMar A/S, Brande, Denmark) with following proximate composition: crude protein 56%, crude fat 12%, ash 9.2%, nitrogen-free extractives 16.5%; gross energy: 20.8 MJ kg^−1^]. Uneaten pellets were removed twice per day (at 7:00 and 14:00), weighed, and subtracted from the recorded feed intake. The daily feeding ration (DFR) was defined 1% of the total tank biomass (adjusted each 28 days). This was done to standardize the feed input across all treatments, avoid waste, and calculate feed conversion efficiency precisely. This restricted feeding regime is the standard procedure in our laboratory for conducting juvenile pikeperch ongrowing trials (26) and ensures that differences in growth and FCR can be attributed to CO_2_ than variable appetite to overfeeding. Settled solids from tanks were removed daily prior to feeding.

### 2.5 Collecting biometric parameters

Prior to any handling during the experimental period, all fish were anesthetized with tricaine mesylate (MS-222; Sigma-Aldrich, St. Louis, USA) at a concentration of 80 mg L^−1^ to minimize stress, following the protocols outlined by (31, 32). At both the start and conclusion of the trial, biometric measurements were performed on anesthetized individuals to determine mean fish size. Total length (TL) and standard length (SL) were measured to the nearest millimeter using a fish measuring board, and body weight (BW) was recorded with a KERN PCB 1000-2 precision balance (Kern & Sohn GmbH, Balingen, Germany).

For these measurements, 50 individuals were sampled from each experimental group, evenly in each tank. All fish across experimental groups were weighed at the beginning and end of the experiment to evaluate coefficient of variation for body weight (CV) and specific heterogeneity variation rate (SHR) (33). The number of fish and mean body weight per tank were documented at these time points and also at 28-day intervals. These data served to calculate actual biomass per tank and to adjust DFR accordingly. The DFR was weighed, and uneaten pellets were subsequently subtracted to determine total daily feed intake (g). Fulton's condition factor (K) of stocked pikeperch was calculated for each tank at the beginning and the end of the experiment within the groups: low, medium and high CO_2_ level. The specific growth rate (SGR), thermal growth coefficient (TGC) (34), survival rate (SR), and feed conversion ratio (FCR) were also calculated for each tank at the end of the experiment. All parameters were calculated as follows:


$$\begin{array}{l} \text{CV }(\%)\;=\text{ SD}/\mu \;\\ \text{SHR }({‰\text{ day}}^{\;-1})\;=\;[\text{ln}(\text{CV final})-\text{ln}(\text{CV initial})]/(\text{D})\;\times 100\\ \text{K }=\;[\text{BW}/{\left(\text{TL}\right)}^{3}]\;\times \;100\\ \text{SGR }(\%\text{ da}{\text{y}}^{-1})=[(\text{lnB}{\text{W}}_{\text{F}}-\text{lnB}{\text{W}}_{\text{I}})/\text{d}]\;\times 100\\ \text{TGC }{(}^{{}^{\circ}}\text{C da}{\text{y}}^{-1})=[\text{B}{\text{W}}_{\text{F}}^{\left(1/3\right)}-\text{B}{\text{W}}_{\text{I}}^{\left(1/3\right)}]/(\text{T }\times \text{ D})\;\times 100\\ \text{SR }(\%)=\text{NF}/\text{NI }\times 100\\ \text{FCR }(\text{g }{\text{g}}^{-1})=\text{TFC}/(\text{FB}-\text{IB}) \end{array}$$

where: SD (g) is standard deviation of body weight, μ (g) is mean of body weight, D (days) is number of experimental days, BW (g) is body weight, TL (cm) is total length, lnBW_F_ and lnBW_I_ is natural logarithm for the final and initial body weight, BW_F_ (g) is final body weight, BW_I_ (g) is initial body weight, T (°C) is temperature of water during experimental days, NF (pcs) final number of harvested fish, NI (pcs) initial number of stocked fish, TGC (g) is total feed consumption, FB (g) is final fish biomass, IB (g) is initial fish biomass.

### 2.6 Plasma biochemical analysis

At the beginning and end of the experimental period, blood samples were collected from two randomly selected individuals (they were not anesthetized in order not to interpolate the measured cortisol levels in the blood of the fish) *per* experimental tank (a total of six samples *per* group). Blood was collected from the caudal vessels (*vena* and *arteria caudalis*) using collection materials treated with an anticoagulant: an aqueous solution of sodium heparin with a concentration of 5,000 IU ml^−1^ (Heparin Léčiva, Zentiva, k. s., Prague, Czech Republic; inj., 1 × 10 ml 50 KU^−1^).

To obtain plasma, blood samples were immediately centrifuged for 15 min 4 000 rpm. Plasma was carefully aspirated using a micropipette with a disposable tip immediately after centrifugation and stored at temperature of −80 °C until analysis.

Biochemical analysis of plasma was performed using a FUJI DRI-CHEM NX500i analyzer (FUJIFILM Europe GmbH, Düsseldorf, Germany). Selected biochemical parameters were determined using reagent slides for following parameters: total protein (TP), albumin (ALB), globulin (GLOB), amylase (AMYL), total cholesterol (TCHOL), glucose (GLU), lipase (LIPA), ammonia (NH3), and triglycerides (TAG). Analysis of cortisol (CORT) was performed using a FUJI DRI-CHEM AU10V fluorescence immunoanalyzer (FUJIFILM Europe GmbH, Düsseldorf, Germany).

### 2.7 Organosomatic indices

Following blood sampling at both the initiation and conclusion of the experiment, fish were euthanized by stunning, immediately terminated by severing spinal cord with guidelines of animal welfare. Selected organs (liver, visceral fat, gonads, kidney) were carefully dissected and weighed using a precision balance (KERN PCB 1000-2; Kern & Sohn GmbH, Balingen, Germany) for the calculation of the hepatosomatic (HSI), fat somatic (FSI), gonadosomatic (GSI) and the kidney somatic (SSI) indices according to formulas:


$$\begin{array}{lll} \text{HSI}\left(\text{\%}\right) & = & \left({\text{W}}_{\text{liver}}/\text{B}\text{W}\right)\times \;100\\ \text{FSI}\left(\text{\%}\right) & = & \left({\text{W}}_{\text{fat}}/\text{B}\text{W}\right)\times \;100\\ \text{GSI}\left(\text{\%}\right) & = & \left({\text{W}}_{\text{gonad}}/\text{B}\text{W}\right)\times \;100\\ \text{KSI}\left(\text{\%}\right) & = & \left({\text{W}}_{\text{kidney}}/\text{B}\text{W}\right)\times \;100 \end{array}$$

where: W_liver_ (g) is weight of the liver, W_fat_ (g) is weight of the visceral fat, W_gonad_ (g) is weight of the gonad, W_kidney_ is weight of the kidney, BW (g) is the body weight.

### 2.8 Fin erosion

Fin condition assessments were conducted at the experiment's beginning and conclusion for all pikeperch, utilizing a single trained observer to mitigate subjective bias. In accordance with the protocol defined by Policar et al. (30), visual evaluations examined the caudal, anal, first and second dorsal, as well as the paired pectoral and ventral fins. Fin erosion was quantified using a standardized four-point scale applicable to pikeperch, where grade 0 denoted negligible erosion (< 5% of fin area), grade 1 indicated minor erosion (5%−30%), grade 2 represented moderate damage (30%−70%), and grade signified severe erosion (>70%).

### 2.9 Statistical analysis

All values are presented as mean ± SD. Statistical analyses were conducted using Statistica software (v.14; TIBCO Software Inc., Palo Alto, CA, USA). The level of significance set at *p* < 0.05. Data normality was assessed using the Shapiro–Wilk test. Depending on the distribution characteristics, either one-way or two-way analysis of variance (ANOVA) was applied. *Post hoc* comparisons were performed using Tukey's honest significant difference (HSD) test to identify pairwise differences. The two-way ANOVA indicated no significant tank effect, confirming homogeneity among replicate tanks.

All parametric tests were preceded by diagnostics of model assumptions. Normality was evaluated using the Shapiro–Wilk test, and homogeneity of variances was assessed using Levene's test (both at α = 0.05). Although one- and two-way ANOVA are generally robust to moderate violations of variance homogeneity, Levene's test (*p* = 0.05) was nevertheless performed for completeness. Prior to deciding on non-parametric tests, we tested commonly applied transformations (log, sqrt, arcsin). These did not simultaneously fulfill the normality (Shapiro-Wilk) and homogeneity (Levene's) assumptions; thus, the Kruskal-Wallis test along with the post-test of Dunn was performed. In addition to the *p*-values, we have included the effect sizes in this study, where applicable (i.e., the partial η^2^ for the ANOVA, while the ε^2^ is used in the Kruskal–Wallis tests). As is well known, the use of an *ad hoc p*-value of 0.05 is problematic, and we have been cautious in interpreting the marginal *p*-values, while relying heavily on the effect sizes in addition to the *p*-values. In this study, we have had three replicate tanks per treatment, while we would recommend that in the future, the replication should be higher in the production-scale trial. Non-parametric analyses were executed in R Studio (R Core Team, v. 4.5.1, 2025) using the stats package (35).

Statistical significance was interpreted as *p* < 0.05 for values between 0.05 and 0.01, and *p* < 0.01 for values below 0.01. This integrated statistical framework ensured rigorous evaluation of group effects and supported robust interpretation of experimental outcomes.

Linear regression analysis was performed on datasets comprising CO_2_ concentrations and average body weight to evaluate dose–response trends. Statistical significance was determined at a threshold (*p* ≤ 0.05).

Although plasma sample size was modest (*n* = 6 per treatment), the inclusion of tank replication and effect-size reporting ensures adequate interpretative robustness. For future prediction-scale trials, additional replication is recommended.

## 3 Results

### 3.1 Production and condition parameters

At the outset of the experiment, pikeperch population exhibited biometric homogeneity. Statistical analyses revealed no significant differences among the Low, Medium, and High groups with respect to initial total length, standard length, body weight, or condition factor (*p* > 0.05). Similarly, the initial coefficient of variation for body weight (CV) did not differ across groups (*p* = 0.861), confirming that all groups commenced the trial with comparable size heterogeneity (Table 2).

**Table 2:** Growth and condition variables of juvenile pikeperch (*Sander lucioperca*) after 105 days (15 weeks) exposed to varying CO_2_ groups: 5 mg L^−1^ = Low; 15 mg L^−1^ = Medium; 30 mg L^−1^= High.

Variable	Low	Medium	High	*F*-statistic	*p*-value
Initial					
TL (mm)	253.91 ± 22.38	254.69 ± 18.10	254.01 ± 19.70	*F*_(2, 447)_ = 3.494	0.187
SL (mm)	218.89 ± 21.86	217.56 ± 17.76	220.12 ± 16.44	*F*_(2, 447)_ = 3.724	0.143
BW (g)	120.65 ± 29.70	115.65 ± 26.64	116.83 ± 28.35	*F*_(2, 447)_ = 1.282	0.278
K	1.12 ± 0.07	1.11 ± 0.09	1.11 ± 0.08	*F*_(2, 447)_ = 0.674	0.613
CV (%)	24.12 ± 0.00	25.16 ± 0.02	24.25 ± 0.03	*F*_(2, 6)_ = 0.153	0.861
Final					
TL (mm)	316.15 ± 29.02^*a*^	318.79 ± 23.40^*a*^	306.75 ± 27.59^*b*^	*F*_(2, 312)_ = 5.864	0.003
SL (mm)	275.74 ± 26.18^*a, b*^	281.90 ± 22.37^*a*^	269.30 ± 25.88^*b*^	*F*_(2, 312)_ = 6.739	0.001
BW (g)	297.34 ± 69.60^*a*^	250.02 ± 69.62^*b*^	232.93 ± 60.81^*a*^	*F*_(2, 312)_ = 26.187	≥0.001
K	1.31 ± 0.09^*a*^	1.19 ± 0.32^*b*^	1.16 ± 0.12^*b*^	F_(2, 312)_ = 15.119	≥ 0.001
CV (%)	28.42 ± 0.02	22.33 ± 0.04	26.17 ± 0.03	F_(2, 6)_ = 2.001	0.216
SHR	127.63 ± 7.07	153.44 ± 18.22	134.40 ± 10.67	F_(2, 6)_ = 2.167	0.196
SGR (% day^−1^)	0.60 ± 0.04	0.44 ± 0.07	0.49 ± 0.05	F_(2, 6)_ = 4.524	0.063
TGC (°C day^−1^)	0.18 ± 0.01	0.15 ± 0.02	0.13 ± 0.02	F_(2, 6)_ = 4.406	0.066
FCR (g g^−1^)	1.09 ± 0.03^*b*^	1.54 ± 0.26^*a*^	1.82 ± 0.35^*a*^	F_(2, 6)_ = 1.311	0.048
SR (%)	99.33 ± 0.94	98.67 ± 1.89	96.00 ± 4.32	F_(2, 6)_ = 0.808	0.489

^a, b^Values within the same row sharing different superscript letters differ significantly (*p* < 0.05).

Values expressed as mean ± SD.

BW, body weight; CV, coefficient of variation of body weight; FCR, feed conversion ratio; K, Fulton's condition factor; SGR, specific growth rate; SHR, specific heterogeneity rate; SL, standard length; SR, survival rate; TL, total length; TGC, thermal coefficient rate.

The ANOVA effect sizes indicated a medium-to-large impact of CO_2_ on final body weight (*F*_(2, 312)_ = 26.187; η*p*^2^ = 0.144), a medium effect on Fulton's condition factor (K) (*F*_(2, 312)_ = 15.119; η*p*^2^ = 0.088), and a small-to-medium effect on total length (*F*_(2, 312)_ = 5.864; η*p*^2^ = 0.037).

**Figure 1:**
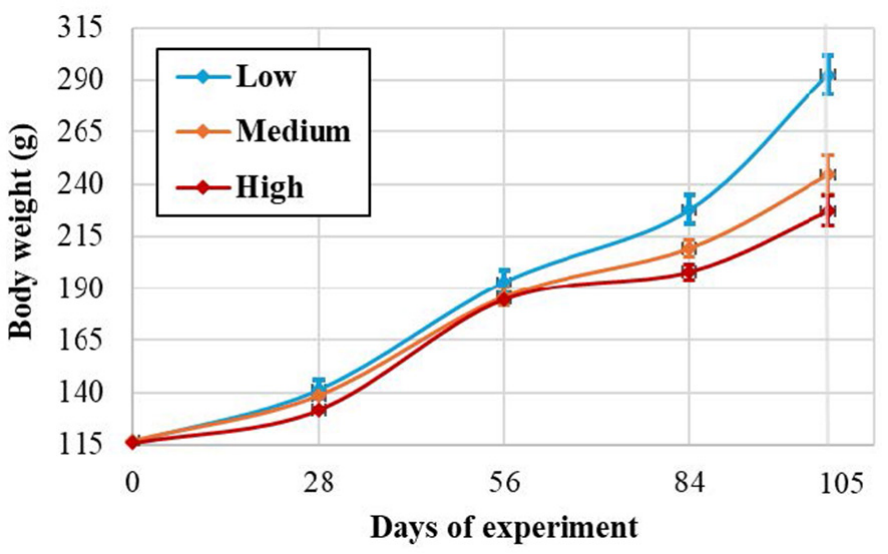
Correlation of pikeperch (*Sander lucioperca*) average body weight and CO_2_ groups: 5 mg L^−1^ = low; 15 mg L^−1^ = medium; 30 mg L^−1^= high. Evaluated on experimental days 0, 28, 56, 84, and 105. Each point represents with standard error of the mean.

By the conclusion of the experiment, exposure to elevated CO_2_ concentrations produced significant effects on somatic growth. Final body weight was strongly influenced by group (*F*_(2, 312)_ = 26.187, *p* < 0.001). *Post-hoc* comparisons demonstrated that individuals in the Low group attained the greatest final body weight (297.34 ± 69.60 g), significantly exceeding that of the Medium (250.02 ± 69.62 g) and High (232.93 ± 60.81 g) groups (Table 2). The analysis showed a significant negative linear relationship between CO_2_ concentration and average fish weight, which gradually increased during the experiment (Figure 1). With increasing CO_2_ levels (Low, Medium, High), there was a systematic decrease in average individual weight, with the correlation being the strongest at the end of the experiment (*p* = 0.002; r^2^ = 0.779). Longitudinal growth followed a similar pattern, with total length differing significantly among groups (*p* = 0.003). Fish in the High group exhibited shorter lengths (306.75 ± 27.59 mm) compared to the Medium group (318.79 ± 23.40 mm), whereas the Low group did not differ significantly from the Medium group (Table 2).

Fish condition, assessed by K, was likewise affected by CO_2_ exposure (*F*_(2, 312)_ = 15.119, *p* < 0.001). The Low group maintained the highest condition factor (1.31 ± 0.09), significantly greater than both the Medium (1.19 ± 0.32) and High (1.16 ± 0.12) groups (Table 2). Despite reductions in growth and condition under elevated CO_2_, size heterogeneity at the end of the trial remained statistically indistinguishable among groups, as indicated by CV (*p* = 0.216) and the specific heterogeneity rate (SHR; *p* = 0.196; Table 2).

Feed efficiency was also significantly influenced by group (*p* = 0.048). The Low group exhibited the most efficient feed utilization, with a feed conversion ratio (FCR) of 1.09 ± 0.03, significantly lower than values observed in the Medium (1.54 ± 0.26) and High (1.82 ± 0.35) groups (Table 2). Growth indices, including the specific growth rate (SGR), declined with increasing CO_2_ concentrations (from 0.60 % day^−1^ in the Low group to 0.49 % day^−1^ in the High group), though these differences approached but did not reach statistical significance (Low: *p* = 0.063; High: *p* = 0.066; Table 2). Survival rate remained consistently high across groups (96.00%−99.33%), with no significant differences detected (*p* = 0.489), indicating that the tested CO_2_ concentrations were sublethal (Table 2).

### 3.2 Plasma biochemical parameters

Several plasma biochemical indices exhibited significant responses to CO_2_ exposure. For plasma biochemistry, effects were large for total protein (F_(2, 15)_ = 6.146; η*p*^2^ = 0.450), albumin (F_(2, 15)_ = 8.611; η*p*^2^ = 0.534), ammonia (F_(2, 15)_ = 7.367; η*p*^2^ = 0.495) and cortisol (F_(2, 13)_ = 4.648; η*p*^2^ = 0.417), underscoring biologically meaningful differences despite the conservative interpretation of *p*-values. Total protein (TP) concentrations were significantly affected (*F*_(2, 15)_ = 6.146, *p* = 0.011), with fish in the High group (35.50 ± 6.06 g L^−1^) displaying markedly lower TP levels compared to those in the Low (44.33 ± 5.68 g L^−1^) and Medium (44.83 ± 3.43 g L^−1^) groups (Table 3). Albumin (ALB) and globulin (GLOB) concentrations followed similar trends, with significantly reduced values in the High group (ALB: 6.17 ± 1.47 g L^−1^, *p* = 0.003; GLOB: 29.33 ± 4.68 g L^−1^, *p* = 0.018) relative to the lower CO_2_ groups (Table 3). Stress and excretory markers were likewise significantly altered. Plasma ammonia (NH_3_) concentrations were highest in the High group (1672.50 ± 156.37 μmol L^−1^), significantly exceeding those in the Low and Medium groups (*p* = 0.006). Cortisol (CORT) levels also varied significantly (*F*_(2, 13)_ = 4.648, *p* = 0.030), with the High group exhibiting the greatest concentrations (574.33 ± 379.87 nmol L^−1^), compared to the Medium group (101.32 ± 116.81 nmol L^−1^) and Low group (244.32 ± 167.85 nmol ml^−1^; Table 3).

**Table 3:** Plasma biochemical parameters in blood plasma of juvenile pikeperch (*Sander lucioperca*) after 105 days (15 weeks) exposed to different CO_2_ groups: 5 mg L^−1^ = low; 15 mg L^−1^ = medium; 30 mg L^−1^= high.

Parameter	Low	Medium	High	*F*-statistic	*p*-value
TP (g L^−1^)	44.33 ± 5.68^*a*^	44.83 ± 3.43^*a*^	35.50 ± 6.06^*b*^	*F*_(2, 15)_ = 6.146	0.011
ALB (g L^−1^)	8.33 ± 1.21^*a*^	8.83 ± 0.75^*a*^	6.17 ± 1.47^*b*^	*F*_(2, 15)_ = 8.611	0.003
LIPA (μkat L^−1^)	0.62 ± 0.12	0.65 ± 0.05	0.56 ± 0.05	*F*_(2, 15)_ = 1.704	0.215
AMYL (μkat L^−1^)	24.03 ± 9.07	20.62 ± 1.35	26.37 ± 4.07	*F*_(2, 15)_ = 1.495	0.256
GLU (mmol L^−1^)	4.75 ± 0.73	4.73 ± 1.40	3.85 ± 0.84	*F*_(2, 15)_ = 1.495	0.256
ALT (μkat L^−1^)	0.66 ± 0.57	0.37 ± 0.23	0.83 ± 0.67	*F*_(2, 14)_ = 1.058	0.373
AST (μkat L^−1^)	1.05 ± 0.39	1.25 ± 0.74	1.20 ± 1.01	*F*_(2, 15)_ = 0.119	0.888
NH3 (μmol L^−1^)	1,236.33 ± 290.52^*b*^	1,201.33 ± 243.98^*b*^	1,672.50 ± 156.37^*a*^	*F*_(2, 15)_ = 7.367	0.006
CORT (nmol L^−1^)	244.32 ± 167.85^*b*^	101.32 ± 116.81^*b*^	574.33 ± 379.87^*a*^	*F*_(2, 13)_ = 4.648	0.030
GLOB (g L^−1^)	36.00 ± 4.52^*a*^	36.00 ± 2.83^*a*^	29.33 ± 4.68^*a*^	*F*_(2, 15)_ = 5.305	0.018

^*a, b*^Values within the same row sharing different superscript letters differ significantly (*p* < 0.05).

Values expressed as mean ± SD.

ALB, albumin; ALT, alanine aminotransferase; AMYL, amylase; AST, aspartate aminotransferase; CORT, cortisol; GLOB, globulin; GLU, glucose; LIPA, lipase; NH3, ammonia; TP, total protein.

### 3.3 Organosomatic indices

In contrast to somatic growth, the relative proportions of internal organs were not significantly affected by CO_2_ exposure. The hepatosomatic index (HSI) ranged from 1.35 ± 0.25 in the Low group to 1.62 ± 0.20 in the Medium group, with no statistically significant differences detected (*F*_(2, 15)_ = 1.478, *p* = 0.259; Table 4). Likewise, the fat somatic index (FSI; *p* = 0.455), gonadosomatic index (GSI; *p* = 0.317), and kidney somatic index (KSI; *p* = 0.438) did not vary significantly among groups (Table 4). These findings indicate that, although elevated CO_2_ concentrations suppressed overall somatic growth, they did not result in disproportionate hypertrophy or atrophy of the liver, gonads, kidney, or visceral fat deposits relative to body size.

**Table 4:** Organosomatic indices of juvenile pikeperch (*Sander lucioperca*) in different groups at the beginning (initial) exposed to different CO_2_ groups after 105 days (15 weeks): 5 mg L^−1^ = low; 15 mg L^−1^ = medium; 30 mg L^−1^= high.

Index	Initial	Low	Medium	High	*F*-statistic	*p*-value
HSI (%)	0.66 ± 0.24	1.35 ± 0.25	1.62 ± 0.20	1.45 ± 0.34	*F*_(2, 15)_ = 1.478	0.259
FSI (%)	0.79 ± 0.38	1.43 ± 0.62	1.96 ± 0.60	1.68 ± 0.86	*F*_(2, 15)_ = 0.830	0.455
GSI (%)	0.04 ± 0.02	0.04 ± 0.05	0.25 ± 0.40	0.15 ± 0.05	*F*_(2, 15)_ = 1.240	0.317
KSI (%)	0.04 ± 0.01	0.08 ± 0.08	0.11 ± 0.06	0.13 ± 0.05	*F*_(2, 15)_ = 0.872	0.438

Values expressed as mean ± SD.

FSI, fat somatic index; GSI, gonadosomatic index; HIS, hepatosomatic index; KSI, kidney somatic index.

### 3.4 Fin erosion

**Figure 2:**
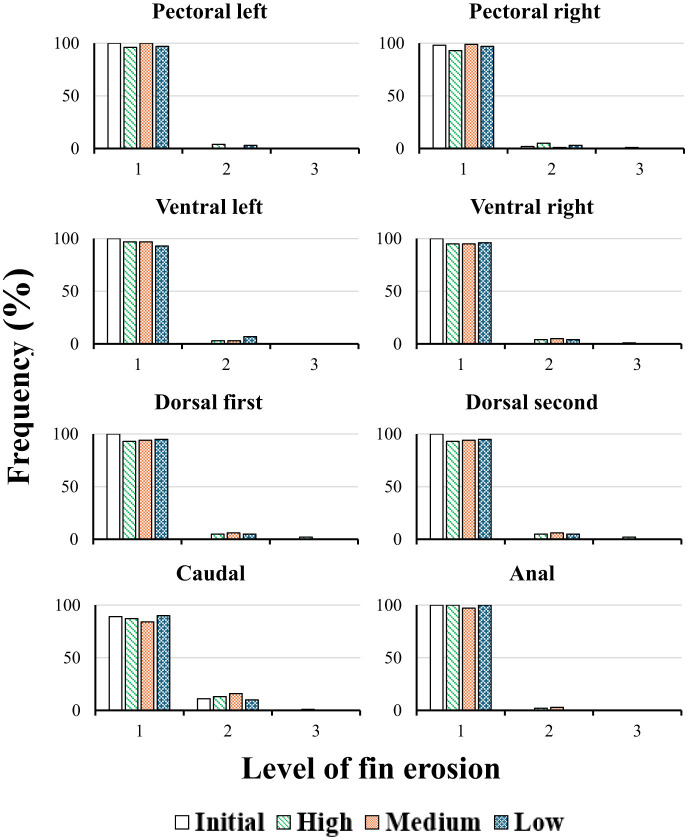
Frequency distribution of fin erosion levels in juvenile pikeperch (grades 1–3: 1 = < 5% area; 2 = 5%−30%; 3 = 30%−70%) across fins (caudal, anal, first and second dorsal, paired pectoral and ventral) and CO_2_ groups (5 mg L^−1^ = low; 15 mg L^−1^ = medium; 30 mg L^−1^= high) over 105 days. A single trained observer, blinded to treatment, performed visual assessments. Stacked bars show the proportion in each grade per fin and group; numbers above bars indicate percentual frequency distribution.

The evaluation of fin erosion across experimental groups indicated that pikeperch generally maintained high fin integrity irrespective of dissolved CO_2_ concentration. Frequency distributions for the pectoral, ventral, and anal fins demonstrated that most individuals in all groups remained at grade 0, corresponding to the absence of observable erosion. For the left pectoral fin, 100% of fish in the initial population, as well as those in the Medium and Low groups, exhibited no erosion, whereas the High group showed a minor incidence of grade 1 erosion in 4% of individuals. Comparable results were observed for the first and second dorsal fins, where the proportion of fish in grade 0 exceeded 93% across all groups (Table 5; Figure 2).

**Table 5:** Percentage distribution of fin erosion scores (grades 1–3: 1 = < 5% area; 2 = 5–30%; 3 = 30%−70%) in different fins of fish across CO_2_ groups: 5 mg L^−1^ = low; 15 mg L^−1^ = medium. 30 mg L^−1^= high.

Fin	Grade	Initial (%)	High (%)	Medium (%)	Low (%)
Pectoral left	1	100	96	100	97
	2	0	4	3	0
	3	0	0	0	0
Pectoral right	1	98	93	99	97
	2	2	5	1	3
	3	0	1	0	0
Ventral left	1	100	97	97	93
	2	0	3	3	7
	3	0	0	0	0
Ventral right	1	100	95	95	96
	2	0	4	5	4
	3	0	1	0	0
Dorsal first	1	100	93	94	95
	2	0	5	6	5
	3	0	0	2	0
Dorsal second	1	100	93	94	95
	2	0	5	6	5
	3	0	0	2	0
Caudal	1	89	87	90	84
	2	11	13	16	10
	3	0	0	1	0
Anal	1	100	100	97	100
	2	0	0	2	3
	3	0	0	0	0

The caudal fin displayed the highest overall frequency of minor erosion relative to other fin types. Grade 1 erosion was recorded in 11% of the initial population, 13% of the High group, 16% of the Medium group, and 10% of the Low group. Despite these minor variations, the occurrence of more advanced erosion (grades 2–3) was virtually absent across all fin categories and groups (Table 5; Figure 2). These findings suggest that the tested CO_2_ concentrations did not exert a detrimental effect on fin condition.

## 4 Discussion

### 4.1 Growth and condition of fish

Our data extends these findings to juvenile pikeperch, over longer production periods, and under conditions of RAS, along with the previous results (8). In percids, including pikeperch, maintenance energy requirements and protein–energy utilization are sensitive to environmental drivers (e.g., CO_2_, temperature, oxygen). Our findings align with species-specific energetics reported for ongrowing pikeperch and complement prior short-term CO_2_ exposures in the species, supporting the view that juvenile pikeperch exhibit limited tolerance margins once dissolved CO_2_ approaches the upper recommended range (8, 15, 26, 27).

Across salmonids, controlled RAS trials also show linear declines in growth as dissolved CO_2_ rises (post-smolts grew best at ≤ 12 mg L^−1^), with stepwise impairments above that threshold, which suggesting optimal performance windows are rather narrow when biomass loading and carbonate chemistry push systems toward chronic hypercapnia (6, 7, 21).

Mechanistically, chronic hypercapnia imposes respiratory acidosis that fish partially compensate via branchial ${\text{HCO}}_{3}^{-}$/Cl^−^ exchange and renal adjustments. The compensation is typically incomplete beyond modest pCO_2_, depressing extracellular pH and constraining aerobic performance and growth. Juveniles may be especially vulnerable because of higher mass-specific metabolic rates and potentially limited acid-based compensation capacity relative to adults (18, 19).

Feed conversion ratio (FCR) deteriorated markedly from 1.09 ± 0.03 (Low group) to 1.82 ± 0.35 (High group), while SGR and TGC trends trailed toward significance. This pattern consistent with subclinical energetic inefficiencies (fish still grow but require more feed per unit biomass). In the Nile tilapia (*Oreochromis niloticus*), hypercapnia and combined hypoxia/hypercapnia reduced voluntary feed intake and feed utilization and can alter digestive physiology (including enzyme activities and gut endocrine signaling), thereby uncoupling intake from accrual (36).

Part of FCR penalty likely reflects osmo-respiratory trade-offs, where hypercapnic fish hyperventilate and devote ion-regulatory work to acid-base recovery. This process can siphon energy from growth and digestion even when standard metabolic rate itself is not increased (23).

The deterioration in FCR with increasing CO_2_ is consistent with subclinical energy expenditure, but alternatively may reflect changes in feeding behavior, feed distribution uniformity, or social interaction in chronically hypercapnic tanks (6, 8, 18, 21, 37). Although CO_2_ was measured three times daily and setpoints were maintained with manual corrections, production RAS may fluctuate in the short term around feed and biomass peaks. We recommend continuous recording or simple summary metrics (e.g., daily range, % time within ±2 mg L^−1^) in future studies.

Despite growth suppression, all organosomatic parameters (HSI, FSI, GSI, and KSI) did not differ among groups, and survival remained high (96–99.3%) across all groups. This indicates CO_2_ levels were sublethal over the 105-day period and did not provoke disproportionate organ hypertrophy/atrophy. In salmonids reared across graded CO_2_, marked effects can be subtle and tissue-specific (e.g., epidermal/dermal thinning without gross organosomatic shifts), again emphasizing that growth and biochemical markers may be earlier or more sensitive indicators than gross morphology (6).

### 4.2 Plasma biochemistry

Biochemical profiles reinforce the interpretation of chronic stress at 30 mg L^−1^ CO_2_. Total protein, albumin, and globulins were significantly lower in the High group, a pattern compatible with reduced protein accretion and possible immune-nutritional impacts. Comparable proteomic work under elevated CO_2_ has shown up-regulation of immune-related proteins and increased energy-metabolism proteins in fish gills, suggesting reallocation of resources and altered homeostasis under acid–base load (38).

Concurrently, plasma ammonia and cortisol were highest in the High CO_2_ group. Hypercapnia disrupts tight acid–base control and can inhibit branchial ammonia excretion. Kidneys then play a larger accessory role yet are themselves under acid–base pressure (39). Chronic hypercapnia and hyperoxia are also recognized risk factors for nephrocalcinosis through complex renal compensations, which underscoring why keeping CO_2_ low is a welfare and health imperative in RAS-based systems (5). Elevated cortisol concentrations in the High CO_2_ group further confirm activation of the hypothalamic-pituitary-interrenal axis, indicative of chronic stress and its associated energetic costs (40). In contrast, digestive enzyme activities (lipase, amylase) and glycemia remained unaffected, implying that hypercapnia primarily perturbs nitrogen metabolism and acid–base homeostasis rather than carbohydrate digestion. The significantly elevated plasma ammonia in the High group reflects disrupted branchial ammonia excretion, consistent with the toxic accumulation that occurs when diffusion and transporter-driven pathways are impaired under acid-base stress (5). Ammonia is known to be highly toxic in fish if retained, causing biochemical and structural damage that reduces growth and welfare (4, 5, 41–43). Collectively, these biochemical shifts emerge as sensitive indicators of sublethal CO_2_ stress, preceding overt morphological changes and reinforcing the need for stringent CO_2_ control to safeguard welfare and performance in RAS-based pikeperch culture. Chronic hypercapnia also puts a constant acid-base demand on the body. In addition to branchial ion exchange, the kidneys play a vital role in reabsorbing bicarbonate and excreting ammonia; branchial excretion of NH_3_/${\text{NH}}_{4}^{+}$ is impaired at high pCO_2_ levels, which puts a greater demand on renal excretion to compensate for acid-base disturbances. This is evidenced by the simultaneous increases in plasma ammonia and cortisol levels at 30 mg L^−1^. Ammonia is a product of impaired excretion and metabolism, while cortisol is a product of HPI activation with subsequent effects on protein metabolism and growth allocation patterns. These findings, combined with the observed decrease in plasma proteins, support a reallocation of energy from somatic growth to ion-regulatory and HPI responses (5, 16, 18, 38–40).

### 4.3 Fin erosion

Fin erosion was generally low and not dependent on CO_2_ tested concentration. There was only minor grade-1 erosion sporadically across groups, with caudal fin most affected (grade 2–3 absent). This contrasts with reports that certain intensive rearing systems or fish species such as Atlantic salmon (*Salmo salar*), can show increased integumentary changes at elevated CO_2_ or under other intensive rearing stressors, e.g., higher water velocities, tank hydraulics (6). Results of this study suggest that used juvenile pikeperch density, hydrodynamics and tested CO_2_ concentrations were not a key driver of fin erosion.

### 4.4 Long-term implications and resilience

Although the tested CO_2_ range was sublethal over 105 days, reduced growth efficiency and condition at ≥15 mg L^−1^ may carry deferred fitness and economic costs beyond the study period. Juveniles could exhibit partial recovery if CO_2_ is reduced subsequently (e.g., compensatory growth), but chronic acid–base challenges can also have cumulative effects on tissue integrity, mineral balance (e.g., nephrocalcinosis risk) and immune function. Longitudinal trials that follow cohorts across entire ongrowing cycles, with phased CO_2_ regimes and recovery periods, are warranted to distinguish reversible from persistent impacts in pikeperch.

To address the remaining uncertainties, we suggest the following: (i) life stage comparisons (from early juveniles to market size) and biomass density gradients to define tolerance windows; (ii) factorial designs combining CO_2_ with O_2_, T, and TAN/NO_2_ to account for interaction effects relevant to RAS water chemistry; (iii) more tank replicates to enhance power for production-scale endpoints; (iv) integrative omics approaches (transcriptomics and proteomics) to gill-kidney axes and endocrine systems under chronic hypercapnia to relate molecular compensation to whole-animal performance in percids.

## 5 Conclusions

High CO_2_ concentrations in RAS resulted in a significant reduction in the growth rate and condition of juvenile pikeperch, which could be attributed to increased metabolic rates and decreased feed efficiency. The results of this study indicate that higher CO_2_ concentrations (>15 mg L^−1^) could be detrimental to the performance of fish, although survival and physiological parameters and fin conditions (erosion) are unaffected. It is recommended that lower concentrations of CO_2_ below 15 mg L^−1^ be maintained to optimize the growth rate, feed efficiency and physiological condition of pikeperch in RAS.

From a practical perspective, the management of dissolved CO_2_ levels below 15 mg L^−1^ in juvenile pikeperch RAS systems would appear to be an important measure to ensure feed efficiency and growth, with monitoring and effective degassing being key management tools. In future, pikeperch should be investigated to determine their tolerance to high levels of CO_2_ at various ages and group sizes. In addition, through genetic and metabolic profiling, it should be determined exactly how these organisms adapt to chronic hypercapnia.

## Data Availability

The datasets presented in this study can be found in online repositories. The names of the repository/repositories and accession number(s) can be found below: https://doi.org/10.5281/zenodo.18456985.
